# Compliance With Bowel Preparation and Its Influencing Factors in Patients Undergoing Colonoscopy: Cross-Sectional Study

**DOI:** 10.2196/77189

**Published:** 2025-10-21

**Authors:** Huan Jiang, Chuanhui Li, Bing Hu, Yi Mou

**Affiliations:** 1 Department of Gastroenterology and Hepatology, West China Hospital, Sichuan University, 37 Guo Xue Alley, Wuhou District, Chengdu, 610041, China, 86 02885422522; 2 Digestive Endoscopy Medical Engineering Research Laboratory, West China Hospital, Sichuan University, China

**Keywords:** colonoscopy, bowel preparation, bowel preparation quality, compliance, adherence, influencing factors, survey, questionnaire, scale, cross-sectional study

## Abstract

**Background:**

Bowel preparation compliance is an important intervenable factor that affects bowel preparation quality, and improving compliance is an important way to optimize bowel preparation outcomes. Despite its importance, the compliance rate and its influencing factors have not been thoroughly evaluated.

**Objective:**

This study aimed to investigate the overall compliance with bowel preparation instructions in patients undergoing colonoscopy.

**Methods:**

From September 2024 to March 2025, a cross-sectional questionnaire-based study was conducted at West China Hospital of Sichuan University, recruiting 740 participants via convenience sampling. We used an 8-item self-report scale to evaluate compliance with bowel preparation instructions. Items were rated on a 4-point Likert scale (0=completely noncompliant to 3=completely compliant), yielding a total score of 0‐24. Higher scores reflected greater compliance, with ≥95% of the maximum score considered adequate compliance. Univariate analysis and multivariate logistic regression analysis were used to assess factors (age, educational level, knowledge of bowel preparation, satisfaction with the taste of the laxative, physical discomfort during bowel preparation) influencing bowel preparation compliance.

**Results:**

In this study, 42.0% (311/740) of patients demonstrated adequate compliance with bowel preparation instructions. In the univariate analysis, hypertension history, knowledge of bowel preparation, laxative type, satisfaction with the taste of the laxative, anxiety during bowel preparation, and physical discomfort during bowel preparation all had statistically significant influences. Multivariate analysis showed that older age (odds ratio [OR] 2.27, 95% CI 1.16-4.49), higher educational level (OR 3.29, 95% CI 1.41-8.33), adequate knowledge of bowel preparation (OR 1.59, 95% CI 1.14-2.24), satisfaction with the taste of the laxative (OR 2.11, 95% CI 1.48-3.02), and no physical discomfort during bowel preparation (OR 0.45, 95% CI 0.31-0.64) were key factors for adequate bowel preparation compliance.

**Conclusions:**

Personalizing bowel preparation instructions according to patients’ age and education level, and selecting a laxative that suits the patients’ taste preferences when available, are feasible ways to improve compliance with bowel preparation.

## Introduction

As the “gold standard” for the diagnosis of intestinal diseases such as colorectal cancer (CRC), colonoscopy provides direct and accurate visualization of the colonic mucosal surface, enabling early detection, accurate diagnosis, and timely intervention, thereby improving patient outcomes [1-3]. In 2021, the US Preventive Services Task Force recommended that people with risk factors such as a family history of CRC, obesity, inflammatory bowel disease, and smoking should undergo regular colonoscopies [4,5]. Even those without these risk factors should undergo CRC screening starting at the age of 45 years and have a colonoscopy every 10 years [4]. The effectiveness of colonoscopy largely depends on the quality of bowel preparation, with adequate cleanliness being essential for a safe, efficient examination and playing a critical role in clinical outcomes [6-8]. However, studies have reported that 20%‐40% of patients undergoing colonoscopy exhibit inadequate bowel preparation, which falls far below the European Society of Gastrointestinal Endoscopy–recommended adequacy standard of 90% [9-11]. Suboptimal bowel preparation may increase the risk of missed lesions, postprocedural complications, and prolonged procedural duration, and necessitate repeat examinations, leading to additional patient discomfort and financial burden [12,13]. Thus, accurate identification of factors associated with bowel preparation holds significant clinical value for improving preparation quality. Numerous studies have identified multiple influencing factors that influence the quality of bowel preparation, including age, gender, BMI, anxiety level, and patient compliance with preparation protocols [14-20]. Strict compliance with bowel preparation instructions is recognized as a critical determinant of successful preparation. Poor compliance with prescribed requirements, including dietary restrictions, fluid intake limitations, and proper medication timing and dosage, may directly affect cleansing efficacy [19,20]. Current research predominantly evaluated compliance through single-dimensional metrics such as laxative consumption or dietary compliance, failing to holistically evaluate the full-process compliance, which includes dietary management, fluid intake restrictions, medication dosage, timing, and duration of medication use [21,22]. This study aims to evaluate the holistic compliance with bowel preparation instructions among patients undergoing colonoscopy and its multidimensional influencing factors in China. The findings will inform the optimization of personalized patient education strategies and the development of targeted preparation guidance, ultimately enhancing compliance to improve bowel preparation quality and subsequent colonoscopy outcomes.

## Methods

### Study Design

This cross-sectional study was conducted at West China Hospital from September 1, 2024, to March 1, 2025. Convenience sampling was used in this study.

### Participants

The participants of this study were patients who underwent colonoscopy at the Endoscopy Center of West China Hospital, Sichuan University. At the time of scheduling a colonoscopy, standardized bowel preparation instructions were given verbally by the endoscopy unit receptionist, who was trained to provide these instructions as a daily routine. Patients and their relatives were also given a brochure as a reminder of bowel preparation instructions. The day before the colonoscopy, the patient began bowel preparation as instructed, including a low-fiber diet and the administration of a laxative, which was sodium phosphate or polyethylene glycol. Before undergoing the colonoscopy, patients waiting in the endoscopy center were invited to voluntarily and independently complete a mobile-based questionnaire by scanning a QR code. The questionnaire contained 32 single-choice items and required approximately 3‐5 minutes to complete.

The following inclusion and exclusion criteria were used:

- Inclusion criteria: age ≥18 years, adequate understanding and communication abilities, and willingness to participate in the study and to sign the informed consent form
- Exclusion criteria: inability to understand the content of the informed consent form and the questionnaire, and failure to cooperate with the investigation or provide informed consent

### Sample Size

The sample size was calculated using a single population proportion sample size–estimating formula. The formula can be given as n = [(Z_1 – α/2_)^2^ p (1 – p)]/d^2^, where, n is the minimum sample size, Z_1 – α/2_ is at a 99% CI of 2.58, *P* is the assumed adequate compliance with bowel preparation (50%), and d is the margin of error to be tolerated (5%). Using this formula, the estimated sample size is 666. Accounting for a 10% nonresponse rate, a total of 740 patients were needed. It should be noted that the 50% expected proportion was chosen as it provides the largest sample size estimate [23].

### Questionnaire

#### General Information Questionnaire

The questionnaire was designed on the basis of a review of the literature and consultation with experts. It includes information on the following aspects: gender, age, educational level, residence, residency status, hypertension history, diabetes history, family history of CRC or polyps, know anyone with colorectal polyps or CRC other than a first-degree relative, and colonoscopy history.

#### Knowledge of Colonoscopy and Bowel Preparation

Patients’ knowledge of colonoscopy and bowel preparation was measured using three and four questions, respectively. Each question has four response options: 0=no understanding at all, 1=a little understanding, 2=moderate understanding, and 3=complete understanding. Higher scores reflect better knowledge, with a score of 5 or above indicating adequate knowledge of colonoscopy and a score of 7 or above indicating adequate knowledge of bowel preparation.

#### Bowel Preparation Compliance

We designed a scale to assess patients’ compliance with various aspects of bowel preparation instructions. The self-developed scale consists of 8 questions and is scored on a 4-point Likert scale, ranging from “completely noncompliant” to “completely compliant,” with scores ranging from 0 to 3. The total score ranges from 0 to 24 points, with higher scores indicating greater bowel preparation compliance. A score greater than 95% of the total score was defined as adequate compliance and vice versa as inadequate compliance. Prior to the start of the formal study, we validated the questionnaire in a preexperiment that included 50 individuals, with a calculated Cronbach α coefficient of 0.877.

#### Current Bowel Preparation Experience Survey

In this section, we investigated the laxative type used by the patients for current bowel preparation, overall satisfaction with the taste of the laxative, anxiety during the bowel preparation, physical discomfort during the bowel preparation, and clarity of the bowel preparation instructions, with each variable classified as binary.

### Study Variable Definitions

#### Dependent Variable

The dependent variable was the bowel preparation compliance (adequate or inadequate).

#### Independent Variables

The independent variables were gender; age; educational level (elementary school or no education, secondary school, university or higher); residence (urban, rural); residency status (living alone or with family); hypertension history; diabetes history; family history of CRC or polyps; know anyone with colorectal polyps or CRC other than a first-degree relative; colonoscopy history; knowledge of colonoscopy (adequate or inadequate); knowledge of bowel preparation (adequate or inadequate); and current bowel preparation experience, including overall satisfaction with the taste of the laxative (satisfactory or unsatisfactory), anxiety during the bowel preparation, physical discomfort during the bowel preparation, and clarity of the bowel preparation instructions.

### Statistical Analysis

The data were analyzed using R version 4.3.2 (R Foundation for Statistical Computing). Continuous variables were presented as means and SDs, whereas categorical variables were expressed as numbers and percentages or frequencies. Intergroup differences with respect to continuous variables were assessed via Student *t* test, if the data met the normality assumption and variance homogeneity. Otherwise, the nonparametric Mann-Whitney *U* test was used. Intergroup differences with respect to categorical variables were assessed using Pearson *χ*² test or Fisher exact probability test, when appropriate.

Variables with *P*<.10 in the univariate analysis were included in the multivariate logistic regression analysis, and independent factors influencing patients’ compliance with bowel preparation were identified through the “enter” selection method (ie, the totally adjusted model). The Hosmer-Lemeshow goodness-of-fit test was used to assess the fitness of the model, with *P*>.05 indicating an acceptable fit. The multivariate analysis results are expressed using odds ratios and 95% CIs only. A *P*<.05 was regarded as statistically significant.

### Ethical Considerations

The research study received approval from the Ethics Committee of West China Hospital, Sichuan University (ChiCTR2400089364). Prior to conducting the questionnaire, written consent was obtained from all participants. The privacy of the participants was respected. All data in the manuscript were anonymized in accordance with ethical standards, ensuring no personally identifiable information could be discerned. No compensation was provided to participants in this study.

## Results

### Sample Characteristics

This study ultimately included 740 patients undergoing colonoscopy. Among the total sample, more than half (n=416, 56.2%) were female, 70% (n=518) had a university education or higher, and 90.5% (n=670) lived in urban areas. The majority (n=704, 95.1%) had no personal history of diabetes, and 86.5% (n=640) had no family history of CRC or polyps. More than half (n=385, 52.0%) of the patients had no prior colonoscopy. Other variables are listed in Table 1.

**Table 1.** General information of patients undergoing colonoscopy (N=740).

Variables		Patients, n (%)
Gender		
Male		324 (43.8)
Female		416 (56.2)
Age (years)		
18-35		152 (20.5)
36‐49		245 (33.1)
50‐64		260 (35.1)
65‐74		75 (10.1)
≥75		8 (1.1)
Educational level		
Elementary school or no education		35 (4.7)
Secondary school		187 (25.3)
University or higher		518 (70.0)
Residence		
Urban		670 (90.5)
Rural		70 (9.5)
Residency status		
Living alone		45 (6.1)
Living with family		695 (93.9)
Diabetes history		
No		704 (95.1)
Yes		36 (4.9)
Hypertension history		
No		632 (85.4)
Yes		108 (14.6)
Family history of colorectal cancer or polyps		
No		640 (86.5)
Yes		100 (13.5)
Know anyone with colorectal polyps or colorectal cancer other than a first-degree relative		
No		444 (60.0)
Yes		296 (40.0)
Colonoscopy history		
No		385 (52.0)
Yes		355 (48.0)

### Knowledge of Bowel Preparation and Colonoscopy and Current Bowel Preparation Experience of Patients Undergoing Colonoscopy

Table 2 presents colonoscopy patients’ knowledge of bowel preparation and colonoscopy, as well as their experience with the current bowel preparation. Of the 740 patients, a total of 56.4% (n=417) and 46.1% (n=341) had adequate knowledge of bowel preparation and colonoscopy, respectively. More than half (n=391, 52.8%) of the patients were unsatisfied with the taste of the laxative. Anxiety and physical discomfort during bowel preparation were experienced by 43.9% (n=325) and 59.3% (n=439) of patients, respectively.

**Table 2.** Knowledge of bowel preparation and colonoscopy and current bowel preparation experience of patients undergoing colonoscopy (N=740).

Variables		Patients, n (%)
Knowledge of bowel preparation		
Inadequate or poor		323 (43.6)
Adequate or excellent		417 (56.4)
Knowledge of colonoscopy		
Inadequate or poor		399 (53.9)
Adequate or excellent		341 (46.1)
Any bowel discomfort prior to this colonoscopy		
No		350 (47.3)
Yes		390 (52.7)
Current colonoscopy schedule		
Morning		244 (33.0)
Afternoon		309 (41.8)
Evening		187 (25.3)
Laxative type		
Sodium phosphate		246 (33.2)
Polyethylene glycol		494 (66.8)
Satisfaction with the taste of the laxative		
Unsatisfactory		391 (52.8)
Satisfactory		349 (47.2)
Anxiety during bowel preparation		
No		415 (56.1)
Yes		325 (43.9)
Physical discomfort during bowel preparation		
No		301 (40.7)
Yes		439 (59.3)
Clarity of bowel preparation instructions		
No		19 (2.6)
Yes		721 (97.4)

### Univariate Analysis of Factors Influencing Compliance With Bowel Preparation

Of the 740 patients, 311 (42.0%) showed adequate compliance with bowel preparation. Intergroup differences were compared between patients with and without adequate bowel preparation compliance (Table 3). The results indicate that hypertension history (*P*=.01), knowledge of bowel preparation (*P*=.004), laxative type (*P*=.008), satisfaction with the taste and flavor of the laxative (*P*<.001), anxiety during bowel preparation (*P*<.001), and physical discomfort during bowel preparation (*P*<.001) all have statistically significant impacts on patient compliance (Table 3).

**Table 3.** Univariate analysis of bowel preparation compliance in patients undergoing colonoscopy.

Variables		Adequate compliance (n=311)	Inadequate compliance (n=429)	Chi-square (*df*)	*P* value
Gender				0.04 (1)	.84
Male		138 (44.4)	186 (43.4)		
Female		173 (55.6)	243 (56.6)		
Age (years)				9.22 (4)	.06
18‐35		57 (18.3)	95 (22.1)		
36‐49		94 (30.2)	151 (35.2)		
50‐64		115 (37.0)	145 (33.8)		
65‐74		42 (13.5)	33 (7.7)		
≥75		3 (1.0)	5 (1.2)		
Educational level				5.97 (2)	.05
Elementary school or no education		9 (2.9)	26 (6.1)		
Secondary school		72 (23.2)	115 (26.8)		
University or higher		230 (74.0)	288 (67.1)		
Residence				1.00 (1)	.32
Urban		286 (92.0)	384 (89.5)		
Rural		25 (8.0)	45 (10.5)		
Residency status				0.19 (1)	.66
Living alone		17 (5.5)	28 (6.5)		
Living with family		294 (94.5)	401 (93.5)		
Diabetes history				0.02 (1)	.90
No		295 (94.9)	409 (95.3)		
Yes		16 (5.1)	20 (4.7)		
Hypertension history				6.53 (1)	.01^a^
No		253 (81.4)	379 (88.3)		
Yes		58 (18.6)	50 (11.7)		
Family history of colorectal cancer or polyps				0.11 (1)	.74
No		271 (87.1)	369 (86.0)		
Yes		40 (12.9)	60 (14.0)		
Know anyone with colorectal polyps or colorectal cancer other than a first-degree relative				1.52 (1)	.22
No		178 (57.2)	266 (62.0)		
Yes		133 (42.8)	163 (38.0)		
Colonoscopy history				0.04 (1)	.85
No		160 (51.4)	225 (52.4)		
Yes		151 (48.6)	204 (47.6)		
Knowledge of bowel preparation				8.35 (1)	.004^a^
Inadequate or poor		116 (37.3)	207 (48.3)		
Adequate or excellent		195 (62.7)	222 (51.7)		
Knowledge of colonoscopy				1.88 (1)	.17
Inadequate or poor		158 (50.8)	241 (56.2)		
Adequate or excellent		153 (49.2)	188 (43.8)		
Any bowel discomfort prior to this colonoscopy				0.00 (1)	>.99
No		147 (47.3)	203 (47.3)		
Yes		164 (52.7)	226 (52.7)		
Current colonoscopy schedule				4.01 (2)	.14
Morning		90 (28.9)	154 (35.9)		
Afternoon		139 (44.7)	170 (39.6)		
Evening		82 (26.4)	105 (24.5)		
Laxative type				7.13 (1)	.008^a^
Sodium phosphate		86 (27.7)	160 (37.3)		
Polyethylene glycol		225 (72.3)	269 (62.7)		
Satisfaction with the taste of the laxative				50.90 (1)	<.001^a^
Unsatisfactory		116 (37.3)	275 (64.1)		
Satisfactory		195 (62.7)	154 (35.9)		
Anxiety during bowel preparation				21.80 (1)	<.001^a^
No		206 (66.2)	209 (48.7)		
Yes		105 (33.8)	220 (51.3)		
Physical discomfort during bowel preparation				64.60 (1)	<.001^a^
No		180 (57.9)	121 (28.2)		
Yes		131 (42.1)	308 (71.8)		
Clarity of bowel preparation instructions				2.69 (1)	.10
No		4 (1.3)	15 (3.5)		
Yes		307 (98.7)	414 (96.5)		

a *P*<.05.

### Multivariate Analysis of Factors Influencing Bowel Preparation Compliance

Binary logistic regression analysis indicated that age, educational level, knowledge of bowel preparation, satisfaction with the taste of the laxative, and physical discomfort during bowel preparation are independent influencing factors for patient compliance (Table 4). The Hosmer-Lemeshow goodness-of-fit test was not significant, indicating that the model fits the data well (*χ*^2^_8_=5.09; *P*=.75).

**Table 4.** Multivariate analysis of bowel preparation compliance in patients undergoing colonoscopy.

Variables		Odds ratio (95% CI)	*P* value
Age (years)			
18‐35		Reference	—^a^
36‐49		1.04 (0.67-1.64)	.85
50‐64		1.23 (0.76-1.98)	.41
65‐74		2.27 (1.16-4.49)	.02^b^
≥75		0.91 (0.16-4.50)	.91
Educational level			
Elementary school or no education		Reference	—
Secondary school		1.99 (0.85-5.00)	.12
University or higher		3.29 (1.41-8.33)	.008^b^
Hypertension history			
No		Reference	—
Yes		1.56 (0.95-2.56)	.08
Knowledge of bowel preparation			
Inadequate or poor		Reference	—
Adequate or excellent		1.59 (1.14-2.24)	.007^b^
Laxative type			
Sodium phosphate		Reference	—
Polyethylene glycol		1.15 (0.81-1.63)	.44
Satisfaction with the taste and flavor of the laxative			
Unsatisfactory		Reference	—
Satisfactory		2.11 (1.48-3.02)	<.001^b^
Anxiety during bowel preparation			
No		Reference	—
Yes		0.82 (0.57-1.18)	.28
Physical discomfort during bowel preparation			
No		Reference	—
Yes		0.45 (0.31-0.64)	<.001^b^

a Not applicable.

b *P*<.05.

## Discussion

One of our study objectives is to evaluate the compliance rate with bowel preparation of Chinese patients undergoing colonoscopy. The survey results revealed that inadequate bowel preparation compliance among colonoscopy patients was up to 58.0% (429/740), which was significantly higher than the 23.6% previously reported in a study [24]. This may be related to the more multidimensional (including dietary management, fluid intake restrictions, medication dosage, timing, and duration of medication use) and demanding (a score greater than 95% of the total compliance score) assessment of bowel preparation compliance in our study.

Our results showed that patients’ compliance with bowel preparation instructions was related to age, educational level, and knowledge of bowel preparation. Patients in the 65‐74 years age group had higher compliance than younger patients in the 18‐35 years age group. This may be related to the fact that the retired population has more time to read bowel preparation instructions, whereas the younger patients may ignore relevant details due to work pressure and busy routines. Moreover, the busy lifestyle and tendency of young people to eat out may also hinder their strict compliance with the long and complex food list for dietary restriction before colonoscopy [25]. Patients with a higher level of education and better knowledge of bowel preparation are more likely to understand the educational content provided by health care professionals [26,27]. These patients may tend to recognize the importance and benefits of adequate bowel preparation for colonoscopy, thus improving their compliance [24]. In the future, personalized health education content can be developed for patients on the basis of their age and educational level to increase their knowledge and improve compliance. For example, customized instructions with concise content can be designed specifically for young people to address their unique challenges.

In this study, the type of laxative significantly influenced compliance in univariate analysis. However, in multivariate analysis, it no longer emerged as an independent factor, suggesting that the effect of laxative type may primarily influence compliance indirectly through associated experiential factors such as taste satisfaction and physical discomfort. Sodium phosphate and polyethylene glycol are two commonly used clinical laxatives that commonly cause adverse reactions such as bloating, nausea, vomiting, and dizziness [28]. These adverse reactions, as well as poor taste and flavor, may result in patients not being able to take adequate amounts of laxative, leading to poor compliance. Of the 740 patients in this study, more than half (n=391, 52.8%) were unsatisfied with the taste of the laxative used, and close to 60% (n=439) experienced physical discomfort during bowel preparation. A study by Hautefeuille et al [29] also showed that patients described nausea and vomiting as the main reasons for noncompliance with bowel preparation protocols. Therefore, for patients with prior experience of bowel preparation, it is important to inquire about their previous experiences with laxatives when they need to undergo bowel preparation again, and laxatives that are acceptable to the patient in terms of taste should be selected to minimize the occurrence of adverse reactions and improve patient compliance.

There are several limitations of this study. First, this study adopted a convenience sampling method and was conducted in a single tertiary hospital in an urban area, which may have introduced selection bias and led to an overestimation of certain indicators (eg, educational level and health awareness of patients). Future research could expand the study to multiple centers to gain a more comprehensive understanding of patient compliance in different settings. Second, bowel preparation compliance was self-reported, which may introduce recall bias, potentially leading to overestimation or underestimation of actual compliance. In addition, it was assessed using a newly developed scale that has only undergone preliminary validation, and its reliability and generalizability require further confirmation in larger and more diverse populations. Third, this study did not collect results on the quality of patients’ bowel preparation, nor did it explore the correlation between compliance and bowel preparation quality. Lastly, due to the cross-sectional design, it is not possible to establish causal relationships. Overall, these limitations highlight critical areas for future research and improvement in understanding and bowel preparation compliance in patients undergoing colonoscopy.

This study revealed that patients had relatively low compliance with bowel preparation. Independent factors identified included age, educational level, knowledge of bowel preparation, satisfaction with the taste of the laxative, and physical discomfort during bowel preparation. Based on the data from this study, we recommend personalizing the educational content of bowel preparation according to the patients’ age and literacy level, and selecting the type of laxative that the patient is satisfied with when available. Our study provides insights for improving bowel preparation compliance in patients undergoing colonoscopy.

## Abbreviations

CRC: colorectal cancer
OR: odds ratio
